# Discovering chromatin motifs using FAIRE sequencing and the human diploid genome

**DOI:** 10.1186/1471-2164-14-310

**Published:** 2013-05-08

**Authors:** Chia-Chun Yang, Michael J Buck, Min-Hsuan Chen, Yun-Fan Chen, Hsin-Chi Lan, Jeremy JW Chen, Chao Cheng, Chun-Chi Liu

**Affiliations:** 1 Institute of Molecular Biology, National Chung Hsing University, Taiwan, ROC; 2 Institute of Genomics and Bioinformatics, National Chung Hsing University, Taiwan, ROC; 3 Department of Biochemistry and the Center of Excellence in Bioinformatics and Life Sciences, State University of New York, Buffalo, NY, USA; 4 Institute of Biomedical Sciences, National Chung Hsing University, Taiwan, ROC; 5 Agricultural Biotechnology Center, National Chung Hsing University, Taiwan, ROC; 6 Department of Genetics, Geisel School of Medicine at Dartmouth, Hanover, NH, USA; 7 Institute for Quantitative Biomedical Sciences, Norris Cotton Cancer Center, Geisel School of Medicine at Dartmouth, Lebanon, NH, USA

## Abstract

**Background:**

Specific chromatin structures are associated with active or inactive gene transcription. The gene regulatory elements are intrinsically dynamic and alternate between inactive and active states through the recruitment of DNA binding proteins, such as chromatin-remodeling proteins.

**Results:**

We developed a unique genome-wide method to discover DNA motifs associated with chromatin accessibility using formaldehyde-assisted isolation of regulatory elements with high-throughput sequencing (FAIRE-seq). We aligned the FAIRE-seq reads to the GM12878 diploid genome and subsequently identified differential chromatin-state regions (DCSRs) using heterozygous SNPs. The DCSR pairs represent the locations of imbalances of chromatin accessibility between alleles and are ideal to reveal chromatin motifs that may directly modulate chromatin accessibility. In this study, we used DNA 6-10mer sequences to interrogate all DCSRs, and subsequently discovered conserved chromatin motifs with significant changes in the occurrence frequency. To investigate their likely roles in biology, we studied the annotated protein associated with each of the top ten chromatin motifs genome-wide, in the intergenic regions and in genes, respectively. As a result, we found that most of these annotated motifs are associated with chromatin remodeling, reflecting their significance in biology.

**Conclusions:**

Our method is the first one using fully phased diploid genome and FAIRE-seq to discover motifs associated with chromatin accessibility. Our results were collected to construct the first chromatin motif database (CMD), providing the potential DNA motifs recognized by chromatin-remodeling proteins and is freely available at http://syslab.nchu.edu.tw/chromatin.

## Background

Chromatin is comprised of repeating nucleosome units consisting of ~146 base pairs of DNA coiled around an octamer of four core histone proteins (H2A, H2B, H3 and H4) [1]. The chromatin surrounding the actively transcribed genes is relaxed, and importantly, a nucleosome-depleted region (NDR) is observed immediately upstream the transcriptional start site. The presence of a NDR is characteristic of both CpG-rich [2] and CpG-poor [3] promoters where transcription factors (TFs) can approach to facilitate transcription.

Gene regulatory elements are intrinsically dynamic and alternate between inactive and active states through the recruitment of DNA binding proteins, such as chromatin remodelers, that regulate nucleosome stability [4]. The formation of open chromatin, or nucleosome disassembly, and its association with transcriptional activity are an evolutionarily conserved characteristic [5]. To date, FAIRE (Formaldehyde-Assisted Isolation of Regulatory Elements) [6], or FAIRE-seq (concerting with massive parallel sequencing), is extensively used to identify cell-specific chromatin states, and to investigate the relationship between chromatin structures and diseases [7-11]. For example, Waki et al. performed computational motif analysis of the adipocyte-specific FAIRE peaks (open chromatin sites) and discovered an enrichment of a binding motif for nuclear family I (NFI) transcription factors [12]. In addition, Song et al. analyzed FAIRE-seq and DNase-seq data in seven cell lines and identified cell-specific regulatory elements [13]. Those studies take advantage of such technology to further reveal the nature of gene regulation. Nevertheless, the effects of allele-specific variations were not considered, and we believe that they may play important roles in chromatin structures.

Recently, Rozowsky et al. integrated RNA-seq, ChIP-seq and the diploid genome sequence to identify allele-specific TF binding sites [14]. Meanwhile, McDaniell et al. integrated DNase-seq, CTCF ChIP-seq and parent–child trios to identify heritable allele-specific chromatin signatures [15]. However, de novo DNA motifs associated with allele-specific chromatin accessibility have not been reported yet. Therefore, we developed the first method for discovering de novo DNA motifs associated with chromatin accessibility using FAIRE-seq and the diploid genome sequence. We mapped the FAIRE-seq reads to the diploid genome and found differential chromatin-state regions (DCSRs) using heterozygous SNPs. The DCSR pairs represent the locations of imbalances of chromatin accessibility between alleles and are ideal to identify motifs that may directly modulate chromatin accessibility [11].

## Results and discussion

### Identifying DCSRs

**Figure 1:**
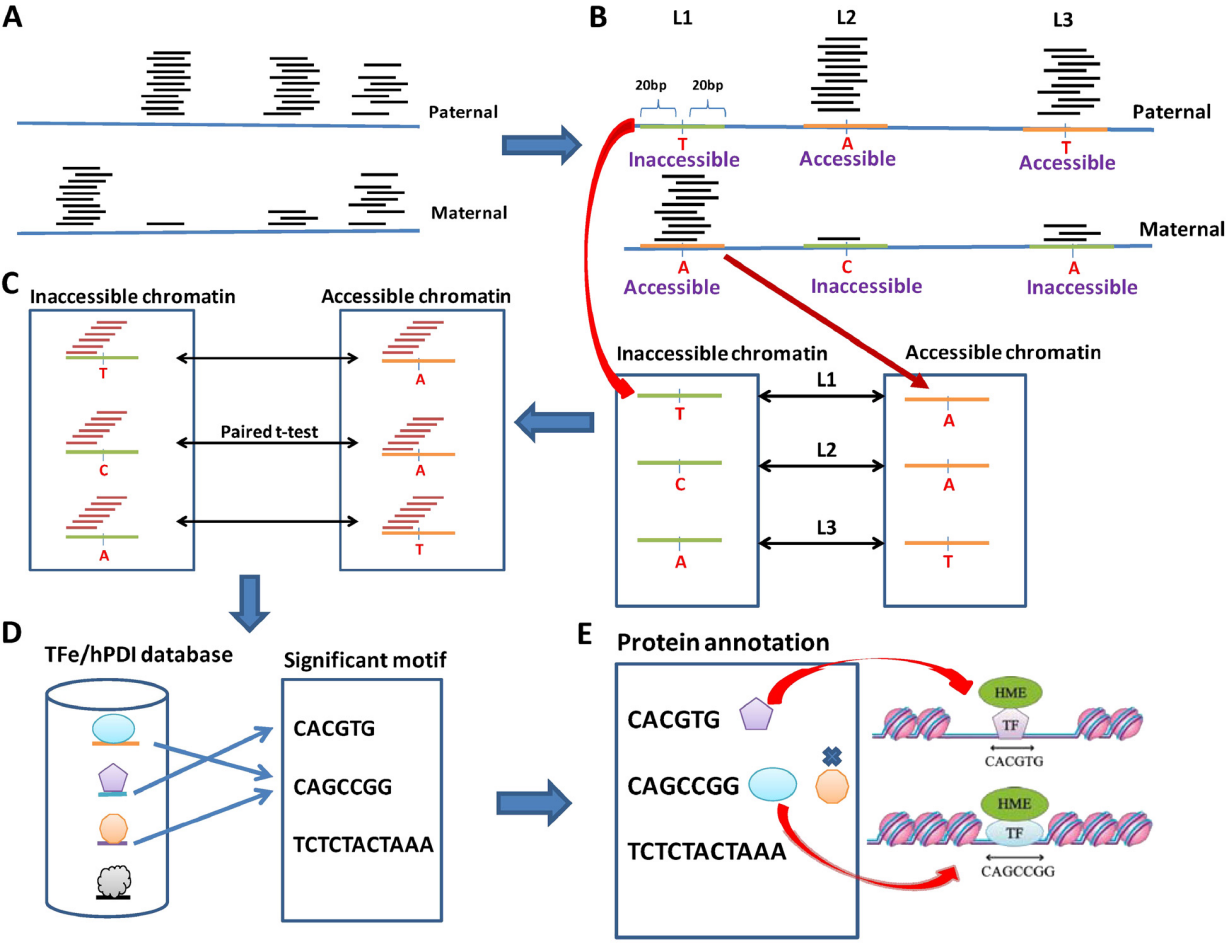
**Overview of the algorithm.** (**A**) To identify accessible and inaccessible chromatin regions, we mapped FAIRE-seq reads to a diploid genome (GM12878) using Bowtie without any mismatch. (**B**) We identified differential chromatin-state regions (DCSRs) and then separated DCSRs into accessible and inaccessible chromatin groups. (**C**) To discover chromatin motifs, we used all combinations of DNA sequences from 6mer to 10mer (motif candidates) to scan all of the DCSRs, each of which has 41 bases in total. For each motif candidate, we calculated occurrence frequency, and then performed paired t-tests between frequency vectors of the inaccessible chromatin group and accessible chromatin group. We selected chromatin motifs with P values < 0.01 by the paired t-tests. (**D**) To annotate the motifs, we used the motif sequences and then performed BLAST alignment against the entire transcription factor binding site sequences in both the TFe and hPDI databases. For each motif, we annotated the motif using the best E-value of alignment and homology > 0.8. **(E)** To provide more accurate annotation, we filtered out the transcript factors that are not expressed (FPKM < 1) by the RNA-seq data (SRP007417).

In this study, we developed a unique genome-wide method to discover DNA motifs associated with chromatin accessibility. We used a publicly available FAIRE-seq dataset with GM12878 cells from UCSC genome browser [6,16] and obtained the corresponding diploid genome sequences from AlleleSeq [14]. The diploid genome allows us to identify binding motifs that differ between alleles and that correspond to differences in chromatin accessibility. The Bowtie tool [17] was used to align FAIRE-seq reads to the genome without any mismatch. Using FAIRE-seq reads and heterozygous SNPs, we can distinguish the reads from paternal or maternal alleles (Figure 1A). Therefore, the chromatin state (accessible or inaccessible) can be determined based on the read depth on heterozygous SNPs (Figure 1B). In other words, the genomic regions with high FAIRE-seq read depth indicate accessible chromatin.

Examining all heterozygous SNPs (~2.3 M heterozygous SNPs) across the genome, we selected the locations with a significant difference in chromatin accessibility between the two alleles. The DCSRs were selected with the following conditions: a SNP was taken into consideration when its read depth had a fold change of >2, and its greater read depth was at least 10 by FAIRE-seq reads. Next, a DCSR was defined to be ±20 bases surrounding such SNP, i.e. 41 bases in total. We used 41 base pairs from both paternal and maternal alleles to build a DCSR pair (Figure 1B).

As a result, we identified a total of 7,829 DCSR pairs in the GM12878 genome, among which 103 are in the promoter regions (TSS −2000 to 0), 2,262 in genes, and 5,464 in the intergenic regions (Table 1). For each pair of DCSRs, we identified the DCSR possessing the higher read depth as accessible chromatin, whereas that possessing the lower read depth as inaccessible chromatin. Figure 1A shows the FAIRE-seq read depth in the diploid genome, and Figure 1B shows that we identified DCSRs and established accessible and inaccessible chromatin groups.

**Table 1:** **Chromatin motifs in genome**-**wide**, **intergenic**, **genic**, **and promoter regions** (**P value** < **0**.**01**)

**Region**	**# DCSRs**	**# Motifs**	**# Annotated motifs**	**Top motif**
Genome-wide	7829	245	163 (66.5%)	CACGTG
Intergenic regions	5464	166	113 (68.1%)	CACGTG
Genic regions	2262	156	100 (64.1%)	CAGGCTGGA
Promoter regions	103	0	N/A	N/A

It is noteworthy that a fully phased diploid genome is important to this study because of the adjacent SNP/indel effect as follows: First, if the distance between the adjacent variations is shorter than the read length, it will affect DCSR detection by changing FAIRE read alignment. Second, if the distance between adjacent variations is < 20 bp, it will affect chromatin motif detection by changing motif frequencies between paternal and maternal alleles.

### Discovering conserved motifs with significant changes in the occurrence frequency among DCSRs

Most of the DCSRs differ in one base from their pairs, implying the SNP directly affects chromatin accessibility (Figure 1B). When a heterozygous SNP occurs in a binding site of a TF, either a histone modification enzyme (HME) itself or a HME-recruiting protein, it may determine the state of chromatin. Thus, SNPs among the DCSRs are essential since they carry the information related to the chromatin accessibility. We thus defined the binding motifs associated with chromatin accessibility as chromatin motifs.

Using DNA 6-10mer sequences to interrogate all DCSRs, we subsequently discovered conserved chromatin motifs with significant changes in occurrence frequency between accessible and inaccessible DCSRs (Figure 1C). Additional file 1 shows the chromatin motifs with occurrence rates in inaccessible and accessible chromatin groups in genome-wide regions (P values < 0.01 by paired *t*-test). There are 1,453 motifs, totally, and 561 (38.6%) of them have higher occurrences in accessible regions than in inaccessible regions. To further eliminate the motifs with low occurrences that might be false positives, we selected motifs that have P values < 0.01 and occurrence rates > 1%. It resulted in 245 genome-wide, 166 intergenic, and 156 genic chromatin motifs (Table 1 and Additional file 2). Since promoter regions only have 103 DCSRs, the number of DCSRs is too small to discover significant motifs. Thus, we did not find any chromatin motif with a P value < 0.01 in promoter regions.

### Annotating chromatin motifs using TF databases

**Figure 2:**
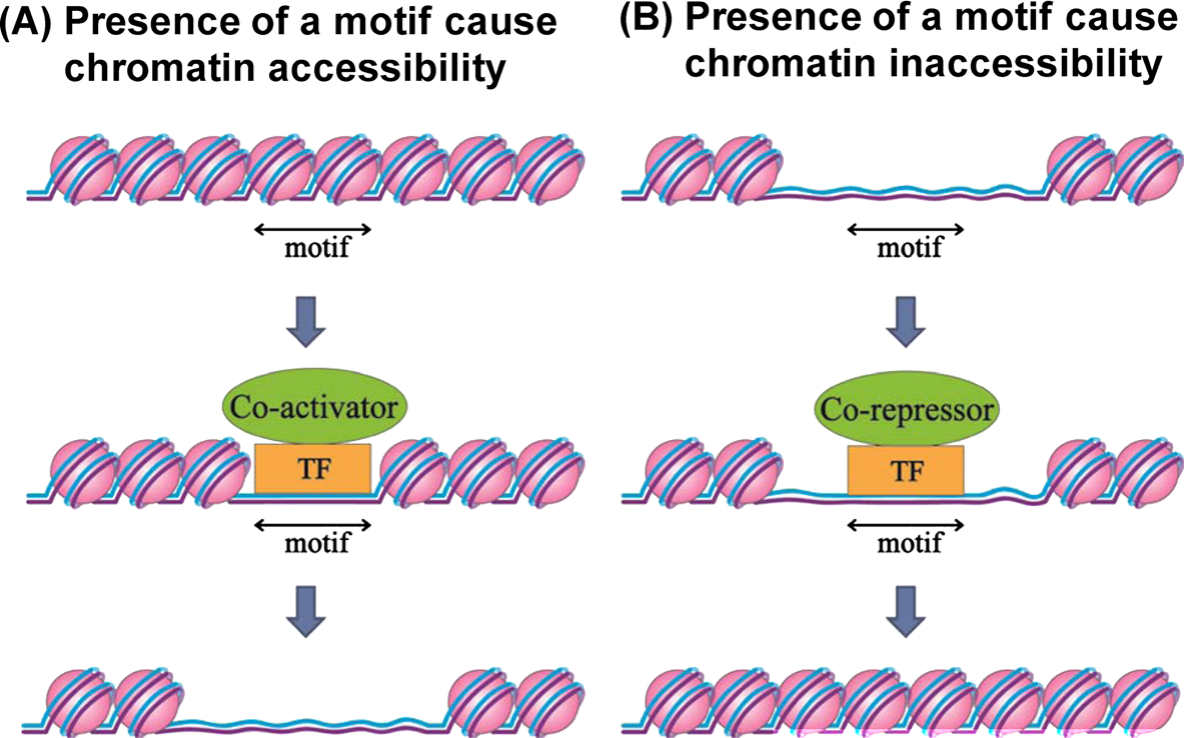
**The hypothesis of chromatin motifs.** (**A**) A chromatin motif in the inaccessible state. The TF may bind to the motif with co-activators and then make the chromatin accessible. (**B**) A chromatin motif in the accessible state. The TF may bind to the motif with co-repressors and then make the chromatin inaccessible.

Grewal and Jia suggested that TFs can recognize specific DNA sequences to nucleate heterochromatin structures [1]. In the same analogy, we proposed that a chromatin motif that can be recognized by a TF may modulate chromatin accessibility (Figure 2). To discover such TFs and to annotate our chromatin motifs, we used two TF databases, the transcription factor encyclopedia (TFe) [18] and the human protein-DNA interactome (hPDI) database constructed using protein microarray assays [19,20], (Figure 1D). As a result, over 60% of motifs can be annotated, and they are listed in Table 1.

To investigate the chromatin motifs and demonstrate the biological significance of the chromatin motifs, we studied the protein annotations of the top ten chromatin motifs in genome-wide, intergenic and genic regions, respectively (Table 2). Within the top ten chromatin motifs, seven genome-wide motifs, six intergenic motifs, and two genic motifs have TF annotations. Surprisingly, most of these annotated motifs have biological reports associated with chromatin remodeling as follows:

**Table 2:** Top ten chromatin motifs and TF annotation

**Region**	**Motif**	**Antisense motif**	**P value**	**Annotation**			**Ref**
				**TFe**	**hDPI logo**	**hDPI motif**	
**Genome**-**wide regions**	CACGTG	CACGTG	7.61E-08		**USF1 USF2**	**MAX USF1 USF2** CREB1 TSNAX ZBTB7A TFEB IRF3 MLX	[21,22,29]
	CTCCTGAC	GTCAGGAG	2.36E-06	**RXR**/**RAR**			[24,25]
	CTGCAG	CTGCAG	3.34E-06			**MAX** IRF3 ZBTB25	[21]
	TTTAGTAGAG	CTCTACTAAA	3.42E-06				
	AGTAGAGA	TCTCTACT	5.71E-06				
	GTGAAACCC	GGGTTTCAC	7.98E-06		**HCFC2** ZNF193 RAB18		[28]
	GGTGAAACCC	GGGTTTCACC	1.23E-05		**HCFC2** ZNF193 RAB18		[28]
	CTCCTGACCT	AGGTCAGGAG	1.33E-05	**RXR**/**RAR**	**RXRA** CREB1 ZNF313 ZNF655	**MAX** USF2 RCOR3 RBM9 GLRX2 ZNF606 CBFA2T3 YBX1 HTATIP IRF3 ESRRA	[24,25]
	AGGTCAGGA	TCCTGACCT	1.41E-05	**RXR**/**RAR**	**RXRA** CREB1 ZNF313 ZNF655	**MAX** USF2 RCOR3 RBM9 GLRX2 ZNF606 CBFA2T3 YBX1 HTATIP IRF3 ESRRA	[24,25]
	TCAGGA	TCCTGA	1.85E-05				
**Intergenic regions**	CACGTG	CACGTG	1.53E-07		**USF1 USF2**	**MAX USF1 USF2** CREB1 TSNAX ZBTB7A TFEB IRF3 MLX	[21,22,29]
	TGTATACA	TGTATACA	1.59E-05				
	ATCACAA	TTGTGAT	2.45E-05	**SOX9**			[25]
	ATGTATACA	TGTATACAT	3.20E-05				
	GGGTTTCAC	GTGAAACCC	3.63E-05		**HCFC2** ZNF193 RAB18		[28]
	GGTGAAACCC	GGGTTTCACC	5.67E-05		**HCFC2** ZNF193 RAB18		[28]
	ATGTATACAT	ATGTATACAT	5.87E-05				
	GGTGAAAC	GTTTCACC	8.17E-05		ZNF193 RAB18		
	CCCGGG	CCCGGG	1.24E-04				
	GGTGAAACC	GGTTTCACC	1.37E-04		ZNF193 RAB18		
**Genic regions**	CAGGCTGGA	TCCAGCCTG	1.58E-06				
	ACTCCAGCCT	AGGCTGGAGT	2.46E-06				
	CTCCAGCCTG	CAGGCTGGAG	2.66E-06				
	TCCAGCCTGG	CCAGGCTGGA	6.96E-06		PIR	**MAX** SIRT2 ZNF34 RFXANK ZBTB7A SCAND2 TSNAX KHDRBS1 ZNF655 TP73 IRF3 NFATC1	[21]
	AGGCTGGA	TCCAGCCT	6.96E-06				
	GGAGGA	TCCTCC	1.16E-05				
	CTCCAGCCT	AGGCTGGAG	1.17E-05				
	TCAGAT	ATCTGA	1.57E-05				
	GGAGTG	CACTCC	1.57E-05				
	CACTCCA	TGGAGTG	2.17E-05	**MEF2A**			[27]

*MAX*: Myc and Mad compete with each other to form a heterodimer with Max [21], and the resulting Myc/Max or Mad/Max protein complex binds to the CACGTG motif through its basic helix-loop-helix leucine zipper domains [22]. The Myc/Max heterodimer is a co-activator that recruits multiple histone acetyl transferase to maintain euchromatin status, whereas the Mad/Max a co-repressor that recruits histone deacetylase (HDAC) to repress transcription. Moreover, Lee et al. identified the genomic binding locations of MYC across 11 different human cell lines using ChIP-seq, and the MYC motif in GM12878 is CCACGTG [23]. This finding is consistent with our top motif CACGTG. *RXR*/*RARA*: RXR/RARA recruits histone deacetylase and represses transcription [24]. In addition, RXR/RARA functions as a local chromatin modulator [25]. *SOX9*: Sox9 interacts with chromatin and activates transcription through the regulation of chromatin modification [26]. *MEF2A*: The interactions between MyoD homodimers and MEF2 proteins may direct HMEs to the chromatin [27]. *HCFC2*: HCFC1 and HCFC2 are the core components of the MLL1 complex, which is a histone methyltransferase acting as a positive global regulator during gene transcription [28]. *USF1*/*USF2*: USF1/USF2 heterodimer recruits HMEs and maintains the chromatin barrier [29].

### Validation

To systematically validate our method, we defined non-differential chromatin-state regions (NDCSRs) by the following conditions: the heterozygous SNPs have a read-depth fold change < 1.5 and the greater allele read depth at least 5 by FAIRE-seq reads. We identified a total of 5,047 pairs of NDCSRs in the GM12878 genome, and then we performed the same framework to obtain significant motifs on NDCSR. Additional file 3 shows the top 100 DCSR and NDCSR motifs with TF annotation. To investigate whether the TF annotation has enrichment with chromatin remodeling, we selected three chromatin-specific GO terms as follows: GO:0016585 chromatin remodeling, GO:0016570 histone modification, and GO:0031490 chromatin DNA binding. In Additional file 3, 29% of DCSR motifs have chromatin-specific annotation and only 8% of NDCSR motifs have chromatin-specific annotation (chi-squared test, P value = 0.0001), suggesting the significant enrichment with chromatin-specific annotation.

To investigate the association of MYC allele-specific binding and differential chromatin-state regions, we applied our method to MYC ChIP-Seq data. The GM12878 MYC ChIP-Seq and control data were downloaded from GEO GSE32883 [23]. The Bowtie tool [17] was used to map ChIP-seq reads to the GM12878 diploid genome to select perfect match and unique mapping tags, which later were fed toChIP-seq processing pipeline [30] to discover narrow peaks with FDR < 0.01. To investigate whether MYC allele-specific binding associates to DCSRs, we calculated the number of DCSRs/NDCSRs with allele-specific or non-allele-specific MYC peaks, and then performed Pearson's Chi-squared test (P value = 0.008). Out result suggests that the differential chromatin-state regions and allele-specific MYC bindings have a significant association (Table 3).

**Table 3:** MYC allele-specific binding associates and DCSR/NDCSR

	**Allele-specific MYC peaks**	**Non-allele-specific MYC peaks**
DCSR	31	36
NDCSR	21	61

### The most significant motif, CACGTG

In Table 2, CACGTG is the most significant motif in both genome-wide (P value = 7.6E-8) and intergenic regions (P value = 1.5E-7). Interestingly, Myc/Max, Mad/Max and USF1/USF2 bind to this motif, and these proteins play the following important roles in chromatin remodeling: the USF1/USF2 heterodimer recruits HMEs to the insulator sites to maintain the chromatin barrier [29]. In mouse studies, Myc and Mad compete with each other to be heterodimerized with Max [21], and the resulting Myc/Max heterodimer or Mad/Max heterodimer binds to the CACGTG motif through its basic helix-loop-helix leucine zipper domains [22]. Myc/Max heterodimer is a co-activator to recruit multiple histone acetyltransferase to maintain euchromatin status, whereas Mad/Max is a co-repressor that recruits histone deacetylase (HDAC) to repress transcription.

We also found that the significant chromatin motifs are not motifs with high occurrence rate. For example, CACGTG has a low occurrence rate of 1.15% in the inaccessible chromatin groups and a low occurrence rate of 0.69% in the accessible chromatin groups (Additional file 2).

## Conclusions

Recently, an increasing number of studies have reported that chromatin accessibility is associated with diseases such as Huntington's disease [31], muscular dystrophy [32], breast cancer [33] and pancreatic cancer [34], reflecting its importance in biology. Our method is the first one to use a diploid genome and FAIRE-seq to discover motifs associated with chromatin accessibility, which leads to the first chromatin motif database (CMD). The CMD provides the potential DNA motifs recognized by chromatin-remodeling proteins.

## Methods

### FAIRE-seq dataset and the diploid genome sequence

FAIRE-seq reads in GM12878 cell line (GSE32883 [23]) were downloaded from the UCSC genome browser [6,16] while diploid genome sequences of GM12878 cell from AlleleSeq [14]. The Bowtie tool [17] was used to map FAIRE-seq reads to the diploid genome without any mismatch. Accessible and inaccessible chromatin regions were identified based on the read depth on the heterozygous SNPs.

### Discovering conserved chromatin motifs

To discover chromatin motifs, we used all combinations of DNA sequences from 6mers to 10mers (~1 M motif candidates) to scan 41-base DCSRs. Since the intergenic and genic regions may have different TFs associated with chromatin remodeling, we performed chromatin motif discovery on the following four type regions: genome-wide, intergenic, genic, and promoter regions (Table 1).

**Figure 3:**
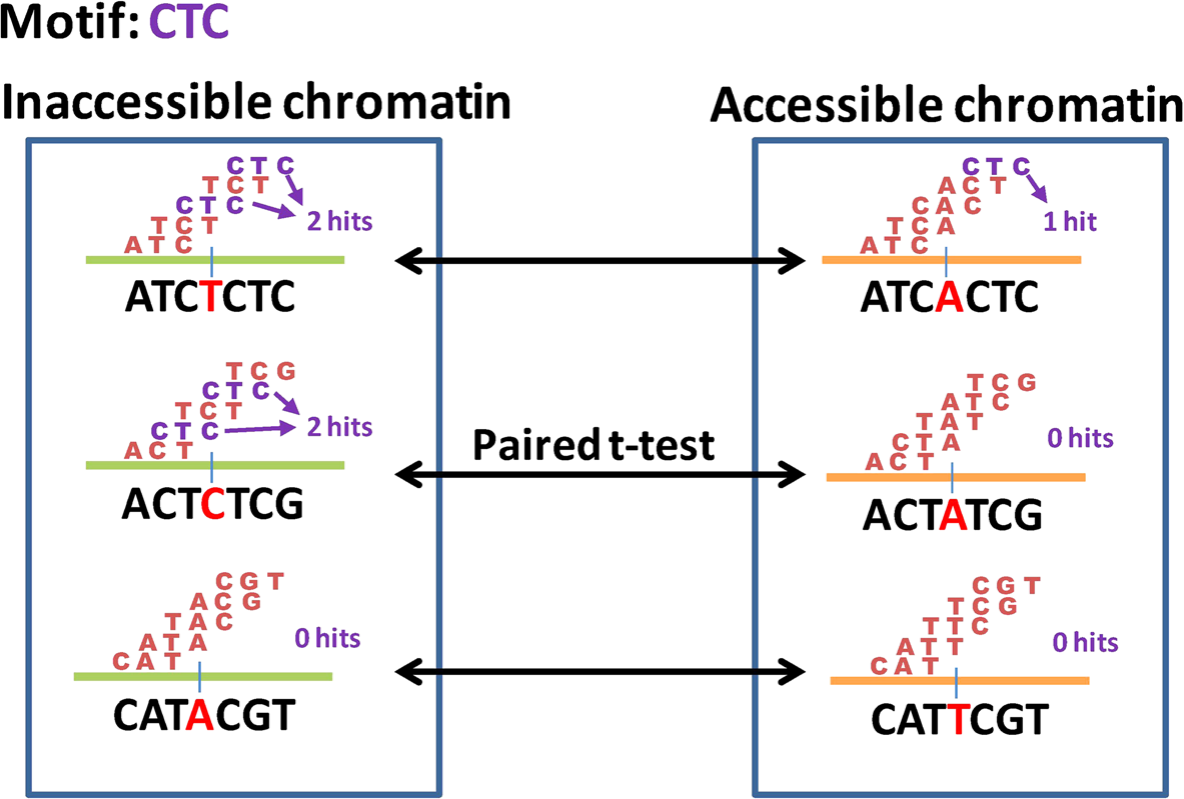
**An example motif CTC.** Assume that a motif candidate is the sequence CTC. We calculated the number of hits (frequency of sequences) among all sliding windows for each DCSR. The frequency vectors of the inaccessible and accessible chromatin groups are [2, 2, 0] and [1, 0, 0], respectively. Next, we performed a paired *t*-test between these two frequency vectors to determine the significance of this motif in terms of differential chromatin states.

Given a motif candidate, we calculated the occurrence frequency using a sliding window on all pairs of DCSRs, and then performed a paired *t*-test between frequency vectors of the inaccessible chromatin group and the accessible chromatin group (Figure 1C). A simple example is illustrated in Figure 3. We assume that the motif has three bases (6 ~ 10 bases in real application); there are three pairs of DCSRs (7829 pairs in real application); and a DCSR has seven bases (41 bases in real application). The frequency vectors of the inaccessible and accessible chromatin groups are [2, 2, 0] and [1, 0, 0], respectively. Furthermore, the paired *t*-test would be performed between these two frequency vectors to select significant motifs.

To select the conserved chromatin motifs associated with differential chromatin states, we applied two conditions as follows: (1) either accessible or inaccessible occurrence rate among DCSRs > 1%; and (2) P values < 0.01 by paired t-test, suggesting a significant frequency change between differential chromatin states.

### RNA-seq data analysis

We downloaded RNA-seq dataset SRP007417 [14] for GM12878 cells from the NCBI Sequence Read Archive [35]. We utilized Bowtie and TopHat [17,36] to map short reads to human genome, and then Cufflinks [37] to estimate the isoform expression level with UCSC KnownGene annotation [38].

### Protein annotation for chromatin motifs

To discover the potential TFs for chromatin modeling, we used two TF databases, the transcription factor encyclopedia (TFe) [18] and the human protein-DNA interactome (hPDI) database [19,20]. We downloaded the TF binding site (TFBS) sequences from the TFe database. Since long TFBS sequences might contain several binding sites, we eliminated any of the TFBS sequences with length >30 bases. We aligned sequences of chromatin motifs to TFBS sequences using BLAST alignment with an E-value threshold of 10. With the TFe database, we annotated a chromatin motif using the best E-value of alignment and homology > 80% between the chromatin motif and TFBS sequences. The best alignment is between a chromatin motif and a short TFBS sequence, which indicates that the TF may bind to the chromatin motif. In addition, we used consensus logos from hDPI to provide TF annotation with consensus motifs.

The hDPI database used a protein microarray-based strategy to build human protein-DNA interactome [20]. Xie et al. selected 460 binding motifs and subsequently constructed double-strand DNA probes with lengths of 6 to 34 bases, in which a binding motif may associate with several TFs. The hDPI database has experimental protein-DNA interaction data for humans identified by the protein microarray assays [20]. The hDPI database provided 460 distinct binding motifs and 201 consensus logos for TFs [20]. With the consensus logos, we may have more precise TF annotation but many chromatin-remodeling TFs are not included in consensus logos such as MAX and HDAC1. Thus, we used both binding motifs and 201 consensus logos to annotate chromatin motifs. With the binding motifs, we annotated a chromatin motif using the best E-value of alignment and homology > 80% between the chromatin motif and binding motif sequences. To annotate chromatin motifs with the hDPI consensus logos, we submitted the chromatin motifs to the hPDI web server (http://bioinfo.wilmer.jhu.edu/PDI/) to obtain match scores, and then annotated the chromatin motifs using a match score > 5 between chromatin motifs and consensus logos (Figure 1D). To provide the confidence level of annotation of hDPI consensus logos, the match score of each annotation is shown in Additional file 2. In addition, to provide the accurate annotation, we filtered out the TFs with fragments per kilobase of transcript per million mapped reads (FPKM) < 1 using the RNA-seq data (Figure 1E).

## Abbreviations

HME: Histone-modification enzyme; FAIRE: Formaldehyde-assisted isolation of regulatory elements; DCSR: Differential chromatin-state region; NDCSR: Non-differential chromatin-state region; CMD: Chromatin motif database; SNP: Single nucleotide polymorphism; TF: Transcription factor; TFe: Transcription factor encyclopedia; hPDI: human Protein-DNA Interactome; FPKM: Fragments Per Kilobase of transcript per Million mapped reads.

## Competing interests

The authors declare that they have no competing interests.

## Authors’ contributions

MJB and CCL designed the algorithm. CCY, MJB, MHC and CCL prepared the manuscript. CCY, HCL, CC and JJWC contributed to the literature study. YFC and CCL constructed the web server. All authors read and approved the final manuscript.

## Supplementary Material

Additional file 1 Chromatin motif lists with occurrence rates in inaccessible and accessible chromatin groups in genome-wide regions. Click here for file

Additional file 2 **Chromatin motif lists with TF annotations in genome-wide, intergenic and genic regions.** We selected motifs from either accessible or inaccessible occurrence rate among DCSRs > 1%. Click here for file

Additional file 3 Top 100 DCSR and NDCSR motifs with TF annotation. Click here for file
